# Two Distinct Superoxidase Dismutases (SOD) Secreted by the Helminth Parasite *Fasciola hepatica* Play Roles in Defence against Metabolic and Host Immune Cell-Derived Reactive Oxygen Species (ROS) during Growth and Development

**DOI:** 10.3390/antiox11101968

**Published:** 2022-09-30

**Authors:** Nichola Eliza Davies Calvani, Carolina De Marco Verissimo, Heather Louise Jewhurst, Krystyna Cwiklinski, Andrew Flaus, John Pius Dalton

**Affiliations:** 1 Molecular Parasitology Laboratory (MPL), Centre for One Health and Ryan Institute, School of Natural Sciences, University of Galway, H91 DK59 Galway, Ireland; 2 Institute of Infection, Veterinary and Ecological Sciences, University of Liverpool, Liverpool L69 3BX, UK; 3 Centre for Chromosome Biology, School of Natural Science, University of Galway, H91 TK33 Galway, Ireland

**Keywords:** antioxidants, excretory–secretory products, helminth, immune defence, oxidative burst, ruminants, trematode, parasite, worm

## Abstract

The antioxidant superoxide dismutase (SOD) catalyses the dismutation of superoxide, a dangerous oxygen free radical, into hydrogen peroxide and molecular oxygen. Superoxide generation during the oxidative burst of the innate immune system is considered a key component of the host defence against invading pathogens. We demonstrate the presence and differential expression of two SODs in *Fasciola hepatica*, a leaderless cytosolic (FhSOD1) and an extracellular (FhSOD3) form containing a secretory signal peptide, suggesting that the parasites exploit these enzymes in distinct ways to counteract reactive oxygen species (ROS) produced by cellular metabolism and immune defences. Both enzymes are highly expressed by the infective newly excysted juvenile (NEJ) stages and are found in abundance in their excretory–secretory products (ES), but only FhSOD1 is present in adult ES, suggesting that the antioxidants have different functions and pathways of secretion, and are under separate temporal expression control during the migration, growth, and development of the parasite. Functionally, the recombinant FhSOD1 and FhSOD3 exhibit similar activity against superoxide to their mammalian counterparts. Confocal immuno-localisation studies demonstrated the presence of FhSOD1 and FhSOD3 on the NEJ tegument and parenchyma, supporting our suggestion that these enzymes are secreted during host invasion to protect the parasites from the harmful oxidative bursts produced by the activated innate immune response. By producing superoxide enzymatically in vitro, we were able to demonstrate robust killing of *F. hepatica* NEJ within 24 h post-excystment, and that the lethal effect of ROS was nullified with the addition of SOD and catalase (the antioxidant enzyme responsible for the dismutation of hydrogen peroxide, a by-product of the SOD reaction). This study further elucidates the mechanism by which *F. hepatica* protects against ROS derived from cellular metabolism and how the parasite could mitigate damage caused by the host’s immune response to benefit its survival.

## 1. Introduction

Fasciolosis, a zoonotic disease of humans and livestock, is caused by infection with the digenean trematodes, *Fasciola hepatica* and *Fasciola gigantica*. The economic impact of fasciolosis on livestock production is expected to exceed USD 3 billion/year [1]. The parasite also infects an estimated 17 million people globally, and 180 million people live in endemic regions where they are at risk of infection [2].

Infection occurs when the mammalian host ingests encysted parasites, metacercariae, carried on plant material or water. The metacercariae excyst in the host’s intestine and the newly excysted juveniles (NEJ) penetrate the intestinal wall and migrate through the abdominal cavity to the liver. Upon reaching the liver, the immature parasites spend 8–12 weeks burrowing through the parenchymal tissue, where they rapidly increase in size and mature. After taking up residence in the bile ducts and gall bladder, the parasites complete their development into egg-laying adults [3]. During this period of migration and growth, the infected host mounts an acute proliferative cellular and humoral immune response to block the parasites and mitigate damage and haemorrhaging caused by their migration. This acute response is characterised by marked eosinophilia, along with the infiltration of macrophages and lymphocytes to the parasite tracks, resulting in the local production of damaging reactive oxygen species (ROS) [4].

Genomic, transcriptomic, and proteomic analyses of several life stages of *F. hepatica* (infective metacercariae, NEJ at 1, 3, and 24 h post-excystment, immature juvenile flukes from 21 days post-infection, and mature adults) have identified many developmentally regulated proteins, thereby shedding light on the complex interactions these parasites have with their hosts throughout the infection process [5,6,7]. The major proteins secreted during these early infection stages include cathepsin-like proteases, protease inhibitors, and a slew of antioxidant enzymes, including superoxide dismutase (FhSOD), peroxiredoxin (FhPrx), thioredoxin (FhTrx), glutathione peroxidase (FhGPx), and glutathione-S-transferase (FhGST) [8]. Together, these proteins are assumed to help tissue invasion, macromolecule digestion, and defend against the onslaught of host-generated ROS [7].

SODs are a class of metalloenzyme antioxidants that, in pathogens, play a role in defence against exogenous ROS produced by the host by catalysing the two-step disproportionation of superoxide anions (O_2_^•-^) into hydrogen peroxide (H_2_O_2_) [9]. Three isoforms of SOD have been described in helminths (worms) and their mammalian hosts based on their localisation and metal co-factors: (1) a cytosolic Cu/Zn SOD, (2) a mitochondrial Mn-SOD, and (3) an extracellular Cu/Zn SOD characterised by the presence of a hydrophobic N-terminal signal peptide [10,11,12,13,14]. To date, only a cytosolic form of Cu/Zn SOD has been described in *F. hepatica* and *F. gigantica* [15,16,17]. However, our interrogation of the existing *Fasciola* spp. genomes revealed the presence of multiple SOD sequences in these parasites, including the cytosolic form, a mitochondrial form, and a novel extracellular SOD possessing a characteristic N-terminal signal peptide sequence. This extracellular SOD is observed at greater protein abundance in the secretome of *F. hepatica* NEJ relative to that of the mature adults, suggesting that it acts as a specialised enzyme protecting the invading parasites against the oxygen-mediated killing mechanisms of their hosts.

Here, we characterised the role of SODs in the defence of *F. hepatica* against ROS via the production of functional recombinant cytosolic (rFhSOD1) and extracellular (rFhSOD3) forms excreted/secreted by the parasite during early invasion. We show that these antioxidants are highly homologous to their mammalian host counterparts, which accounts for their lack of immunogenicity in infected sheep. Specific anti-rFhSOD1 and anti-rFhSOD3 antibodies were produced and used in immuno-localisation experiments to identify the site of enzyme production and secretion in *F. hepatica* NEJ and adults. Finally, we developed an in vitro biological assay that enzymatically produces ROS and demonstrates the susceptibility of NEJ to both superoxide and hydrogen peroxide. This killing of NEJ was obviated via the addition of SOD and catalase, revealing the power of this cascade in defence against ROS during invasion.

## 2. Materials and Methods

### 2.1. Identification and Phylogenetic Analysis of F. hepatica Superoxide Dismutases

The *F. hepatica* SOD gene sequences were identified by BLAST analysis using the previously reported *F. hepatica* SOD sequence (Kim et al., 2000 [16]; AF071229) against the *F. hepatica genome* (WormBase ParaSite Version WBPS16 (WS279): PRJEB6687 and PRJEB25283; Cwiklinski et al., 2015 [5]; FhSOD1: BN1106_s3189B000243; maker-scaffold10x_61_pilon-snap-gene-0.36, FhSOD2: snap_masked-scaffold10x_1664_pilon-processed-gene-0.2, FhSOD3: BN1106_s4478B000037; maker-scaffold10x_713_pilon-snap-gene-0.105). Annotation of the resulting sequences was confirmed using in silico tools (Uniprot, Gene Ontology (GO), and InterProScan).

Homologous trematode SOD DNA sequences were identified and retrieved using BLAST analysis of publicly available genome databases at WormBase Parasite (http://parasite.wormbase.org/index.html Version WBPS16 (WS280), accessed on 7 April 2021). Genomic DNA sequences were imported, manually inspected, translated, and aligned in CLC Main Workbench 21.0.3 (Table S1). Identification of N-terminal signal peptide sequences was carried out using the SignalP and TMHMM plugin (Version 21.0) in CLC Main Workbench (Version 21.0.3). Previously characterised mammalian SOD sequences (*Ovis aries, Bos taurus, Bos indicus*, and *Homo sapiens*) were downloaded from GenBank (National Center for Biotechnology Information, NCBI) (Table S1). Ambiguous regions (i.e., containing gaps and/or poorly aligned) were removed from the resultant amino acid alignment of all mammalian and trematode sequences with Gblocks (v0.91b) using the following parameters: minimum length of a block after gap cleaning: 10; positions with a gap in less than 50% of the sequences were selected in the final alignment if they were within an appropriate block; all segments with contiguous non-conserved positions longer than 8 were rejected; minimum number of sequences for a flank position: 85% [18,19]. The resultant sequence alignment spanned 107 amino acids (Gly 67-Gly173, relative to FhSOD1), containing 43 amino acid sequences, which was submitted for Smart Model Selection (SMS) using PhyML 3.0 (Figure S1) [20]. Evolutionary history was inferred using the maximum likelihood method and the Whelan and Goldman (WAG + G+I) model, with 1000 bootstrap support in MEGA11 [21,22]. Initial tree(s) for the heuristic search were obtained automatically by applying Neighbor-Join and BioNJ algorithms to a matrix of pairwise distances estimated using the JTT model, and then selecting the topology with superior log likelihood value. A discrete Gamma distribution was used to model evolutionary rate differences among sites (5 categories (+G, parameter = 1.3434)). The rate variation model allowed for some sites to be evolutionarily invariable ([+I], 11.21% sites). Amino acid sequence similarity and identity was determined using UniProt ClustalO [23].

### 2.2. Transcriptomic and Proteomic Expression Analysis of FhSOD1 and FhSOD3

Stage-specific *F. hepatica* transcriptome datasets, previously described by Cwiklinski et al. (2015; PRJEB6904) [5] were interrogated to determine the differential transcription of the *FhSOD1* and *FhSOD3* genes, represented as the number of transcripts per million (TPM). *F. hepatica* proteomic datasets were interrogated to determine the FhSOD1 and FhSOD3 protein abundance, represented by the exponentially modified protein abundance index (emPAI) within the somatic proteome and the secreted protein fraction (secretome/ES proteins), using Scaffold (version Scaffold_5.1.2, Proteome Software, Portland, OR, USA). Analysis of the somatic proteome was carried out for the metacercariae, NEJ (3, 24, and 48 h post-excystment) and immature life cycle stages using the data reported by Cwiklinski et al. (2018, 2021) [6,7]. Secretome analysis was carried out on the 24 h NEJ, immature, and adult liver fluke secreted proteins using the data reported by Cwiklinski et al. (2018, 2021) [6,7] and Murphy et al. (2020).

### 2.3. Preparation of Excretory/Secretory (E/S) Products and Somatic Extracts from Adult F. hepatica

Adult liver flukes were collected from the livers of sheep during post-mortem abattoir surveillance in Roscommon, Ireland, as previously described [24]. The parasites were washed with sterile 1× PBS before culturing in RPMI medium (containing 0.1% glucose, 100 U penicillin and 100 mg/mL streptomycin) at a ratio of 1 worm/2 mL at 37 °C and 5% CO_2_. After 2 h, culture media (containing the E/S) was collected and centrifuged at 300× *g* for 10 min and then 700× *g* for 30 min to eliminate large debris. The supernatant was subsequently concentrated using a spin column protein concentrator with a molecular weight cut-off of 3 kDa (Amicon ultra, Merck Millipore, Burlington, MA, USA), aliquoted and frozen at −80 °C prior to use.

Adult somatic proteins were extracted from a frozen adult fluke via homogenisation in 100 µL DPBS followed by centrifugation at 300× *g* for 15 min and then 800× *g* for a further 15 min. The protein concentration in the resultant supernatant of the somatic extract and E/S was measured using the Bradford Protein Assay (Bio-Rad).

### 2.4. Expression and Purification of Functional Recombinant FhSOD1 and FhSOD3 in Escherichia coli

The cytoplasmic (*FhSOD1*) and extracellular (*FhSOD3*, with the signal peptide sequence removed) sequences were codon optimised for expression in *Escherichia coli* and individually cloned into pET-28a(+) vectors with a C-terminal His-tag (GenScript, Piscataway, NJ, USA). The vectors were electro-transformed into kanamycin-resistant ClearColi BL21 (DE3) (ThermoFisher Scientific, Waltham, MA, USA) cells, with transformants selected on LB-Miller + kanamycin (50 μg/mL) agar plates after growth overnight at 37 °C.

Recombinant cells harbouring *FhSOD1* and *FhSOD3* were grown in LB-Miller broth at 37 °C and 180 rpm with 50 μg/mL kanamycin until the OD600 was between 0.7 and 0.8. Isopropyl-β-d-thiogalactopyranoside (IPTG; ThermoFisher Scientific) was added to the culture medium at 0.5 mM to induce protein expression, and cultures were incubated at 21 °C for a further 21 h. A 1 mL aliquot of each culture was removed at T_0_ and T_21_ to monitor protein production. Following centrifugation at 10,000× *g* for 10 min at 4 °C, the recovered bacteria were resuspended in sterile ST buffer (10 mM Tris, 150 mM NaCl, pH 8.0) and stored at −20 °C overnight. Cell pellets were thawed on ice then treated with 10 mg/mL lysozyme for 30 min. After incubation, 750 μL 10% Sodium lauroyl sarcosinate (sarcosyl) was added to the pellets prior to sonication at 70% amplitude with six cycles of 10 s burst/10 s rest on ice.

To recover soluble protein, the supernatant was collected after centrifugation at 15,000× *g* for 30 min at 4 °C, diluted to a final volume of 50 mL in lysis buffer (sodium phosphate buffer, pH 8, 10 mM imidazole) and purified using the Profinia Affinity Chromatography Protein Purification System (Bio-Rad, Hercules, CA, USA) with the corresponding mini profinity IMAC and mini Bio-Gel P—6 desalting cartridges (Bio-Rad). Proteins were eluted into 4 mL of 1× PBS, aliquoted, and stored at –70 °C until use. Protein concentration and purity were verified immediately after purification using the Bradford Protein Assay (Bio-Rad) and by 4–20% SDS-PAGE gels (Bio-Rad) stained with Biosafe Coomassie (Bio-Rad), respectively. To further confirm the expression and purification of the recombinant proteins, Western blots were performed using a monoclonal mouse anti-polyhistidine antibody (1:10,000) (Sigma-Aldrich, St. Louis, MO, USA) as a primary antibody, followed by incubation with a secondary antibody alkaline phosphatase conjugated goat to mouse-anti-IgG diluted 1:5000 (Sigma-Aldrich). The gels and Western blots were visualised using a G:BOX Chemi XRQ imager (Syngene, Bengaluru, India).

The tertiary states of the recombinant FhSOD1 and FhSOD3 proteins were resolved by size-exclusion chromatography (gel filtration) performed on a high performance Superdex 75 10/300 GL (Tricorn) column, with a flow rate of 400 μL/min and eluted into 1× PBS. Three proteins of different molecular sizes were resolved in the column as standards, namely conalbumin (76 kDa), carbonic anhydrase (29 kDa), and aprotinin (6.5 kDa) (GE Healthcare). Upon determination of the retention parameters, rFhSOD1 and rFhSOD3 were added to the column, with 200 μL aliquots of each purification fraction collected and stored at 4 °C for enzyme activity (see below). The 3D structure of the corresponding native FhSOD1 (D915_003308) and FhSOD3 (D915_009739) amino acid sequences was predicted by the AlphaFold Protein Structure Database [25,26].

### 2.5. Analysis of rFhSOD1 and rFhSOD3 Enzyme Activity

The enzymatic activity of the recombinant FhSODs was measured via the adaptation of an existing xanthine oxidase (XOD)-based SOD assay, whereby O_2_^•-^ is enzymatically produced because of the conversion of xanthine to H_2_O_2_ and uric acid, which in turn transforms nitroblue tetrazolium (NBT) to NBT-diformazan dye [27]. To determine the activity of our recombinant enzymes under physiological conditions compatible with the parasite, our assay was conducted at 37 °C in 200 µL assay buffer (1 × PBS; Sigma-Aldrich, 0.1 mM Hypoxanthine; Sigma-Aldrich, 0.1 mM DTPA; Sigma-Aldrich, 2.5 µL/mL tetrazolium salt; Cayman Chemical, Ann Arbor, MI, USA). All other enzymes (native XOD from bovine milk; Roche, used at a final concentration of 8.0 × 10^−^^3^ U/mL), including the standard curve (native bovine erythrocyte SOD, BS; Sigma-Aldrich), were diluted in PBS prior to use. Recombinant proteins and the standard curve (BS) were serially diluted 1:1 in PBS (rFhSOD1 and rFhSOD3: 10–0.3125 μg/mL; BS: 100–3.125 μg/mL, corresponding to 4–0.125 U/mL) and run in duplicate at a final assay volume of 250 μL. The assay was read continuously for 30 min at 450 nm using a microplate reader (PolarStar Omega Spectrophotometer; BMG LabTech, Ortenburg, Germany) immediately after the addition of XOD.

### 2.6. Immune Recognition of FhSOD1 and FhSOD3 in Sera from F. hepatica Experimentally Infected Sheep

Sera were collected from *F. hepatica* experimentally infected sheep and assayed as previously described by Lopez Corrales et al. (2020). The infections were carried out by Agri-Food and Biosciences Institute (AFBI; Belfast, UK) under license from the Department of Health, Social Services and Public by the *Animal (Scientific Procedures) Act* 1986 (License No. PPL 2771; PPL 2801). FhSOD1 and FhSOD3 total IgG antibodies were analysed by ELISA and Western blot according to standard methods. Briefly, for the ELISA, flat-bottom 96-well microtitre plates (Nunc MaxiSorp, Biolegend, San Diego, CA, USA) were coated with rFhSOD1, rFhSOD3, or recombinant *F. hepatica* cathepsin L1 (rFhCL1) (5 μg/mL in 0.05 M carbonate buffer, pH 9.6) and incubated overnight at 4 °C [28]. After incubation in blocking buffer (2% bovine serum albumin in PBS-0.05% Tween-20 (*v*/*v*), PBST, pH 7.4) and washing three times in PBST, sheep sera collected from 10 animals at 0, 3, 7, 11, 15, and 23 weeks post-infection (WPI) was diluted 1:100 in serum dilution buffer (PBS, 0.5% Tween 80, 0.5 M NaCl), added to the antigen-coated wells in triplicate, and allowed to incubate for 1 h at 37 °C. After washing five times, 100 μL/well of HRP-conjugated donkey anti-sheep IgG (ThermoFisher Scientific) diluted 1:50,000 in blocking buffer was added, and the plates were incubated for 1 h at 37 °C. Following five washes, 100 μL/well of 3,3′,5,5′-Tetramethylbenzidine (TMB; Sigma-Aldrich) was added, and the plates were incubated at room temperature (RT) for 4.5 min. The reaction was stopped by the addition of 100 μL/well of 1 M sulphuric acid. The optical density was determined at a wavelength of 450 nm (OD450) in a PolarStar Omega spectrophotometer (BMG LabTech, Ortenburg, Germany).

Western blot analysis was performed using standard methods [29]. Briefly, rFhSOD1 and rFhSOD3 (1 μg/lane) was resolved by electrophoresis in 4–20% precast SDS-PAGE gels and electro-transferred onto nitrocellulose membranes prior to incubation in blocking buffer (5% milk in PBST, pH 7.4) for 1 h at RT. rFhCL1 was used as a positive control at 7 WPI. After washing five times with PBST, the membranes were probed with pooled sera from experimentally infected sheep at 0, 7, and 20 WPI diluted 1:1000 in blocking buffer (2.5% milk in PBST, pH 7.4) for 1 h at RT. After washing five times in PBST, the membranes were incubated with the secondary antibody alkaline phosphatase conjugated donkey-anti-sheep IgG at a 1:10,000 dilution for 1 h at RT. Following a final wash, the immune-reactive bands were visualised using the substrate SigmaFast BCIP/NBT (Sigma-Aldrich).

### 2.7. Production of Specific Antibodies against rFhSOD1 and rFhSOD3

Non-homologous sequences at the N-terminal of FhSOD1 (VMSGSSGVQGTVKFVQESET) and FhSOD3 (NASYSGQIFVNADGNLLTVR) were identified and protein-specific peptides (pFhSOD1 and pFhSOD3) were synthetically produced coupled with ovalbumin and used to immunise rabbits to generate FhSOD1 and FhSOD3-specific antibodies (Anti-pFhSOD1 and Anti-pFhSOD3) (Eurogentec, Seraing, Belgium). In addition, polyclonal antibodies against the purified recombinant rFhSOD1 and rFhSOD3 proteins (Anti-rFhSOD1 and Anti-rFhSOD3) were produced in rabbits (Eurogentec). Anti-rFhSOD1 and anti-rFhSOD3 antibodies were adsorbed against recombinant rFhSOD3 and rFhSOD1, respectively, prior to use to ensure specificity. The reactivity of the anti-pFhSOD1 and pFhSOD3 antibodies and anti-rFhSOD1 and rFhSOD3 polyclonal antibodies was determined by Western blot analysis against 0.05 μg/lane rFhSOD1 and rFhSOD3. Native bovine erythrocyte SOD (BS; 0.05 μg/lane) was used as a negative control. Immuno-detection was conducted as described above, with the following exceptions: membranes were probed with rabbit pre-immune sera, anti-pFhSOD1, anti-pFhSOD3, anti-rFhSOD1 or anti-rFhSOD3 raised in rabbits at a 1:10,000 dilution for 1 h at RT. After washing, the membranes were further probed with the secondary antibody, alkaline phosphatase conjugated goat-anti-rabbit IgG, at a 1:5000 dilution for 1 h at RT. Following final washes, the immune-reactive bands were visualised using the substrate SigmaFast BCIP/NBT (Sigma-Aldrich).

### 2.8. Immuno-Detection of Native FhSOD1 and FhSOD3 in NEJ, Adult Parasites and Their Extracts

Localisation of FhSOD1 and FhSOD3 in NEJ was conducted as follows: *F. hepatica* metacercariae (Italian isolate; Ridgeway Research, St. Briavels, UK) were excysted and cultured in RPMI 1640 medium containing 2 mM L-glutamine, 30 mM HEPES, 0.1% (*w*/*v*) glucose, 2.5 µg/mL gentamycin, and 10% foetal calf serum (ThermoFisher Scientific) for 24 h, as previously described [29]. NEJ were then fixed with 4% paraformaldehyde (PFA) in 0.1 M PBS (Sigma-Aldrich) pH 7.4, for 1 h at RT. After three washes in antibody diluent (PBS containing 0.1% (*v*/*v*) Triton X-100, 0.1% (*w*/*v*) bovine serum albumin and 0.1% (*w*/*v*) sodium azide; AbD buffer), the NEJ were incubated in rabbit pre-immune sera, anti-rFhSOD1, anti-rFhSOD3, or anti-rFhCL3 polyclonal antibodies (used as a non-related positive control) diluted 1:500 in AbD buffer overnight at 4 °C. After three washes in AbD, the NEJ were incubated in a 1:200 dilution of the secondary antibody, fluorescein isothiocyanate (FITC)-labelled goat-anti-rabbit IgG (Sigma-Aldrich) overnight at 4 °C in the dark. To counter-stain muscle tissue, the samples were incubated in AbD containing phalloidin-tetramethylrhodamine isothiocyanate (TRITC) (200 μg/mL) overnight in the dark at 4 °C. The NEJ were then whole-mounted onto slides using 10% glycerol solution containing 0.1 M propyl gallate and visualised under an Olympus Fluoview 3000 laser scanning confocal microscope using a PL APO CS 6 × 0 oil objective lens. Olympus type F immersion oil was used in viewing and all images were taken at room temperature.

Localisation of FhSOD1 and FhSOD3 in adult *F. hepatica* was conducted on parasites collected from the livers of infected sheep during post-mortem surveillance in Roscommon, Ireland. After collection, the parasites were washed in 1 × PBS and fixed in 4% PFA for 4 h at RT. After 4 h, the PFA was removed, replaced with 1 × PBS followed by an incubation for 1 h at RT. This washing process was repeated twice before dehydration in ascending ethanol and subsequent infiltration with JB-4 resin (Sigma-Aldrich EM0100). Serial 0.5 µM sections were mounted onto slides and probed with rabbit pre-immune sera, anti-rFhSOD1, anti-rFhSOD3, or anti-rFhCL1 polyclonal antibodies (used as a non-related positive control) diluted 1:1000 in PBST for 5 h at RT in a humid container. The sections were washed three times in PBST before addition of a 1:1000 dilution of the secondary antibody, FITC-labelled goat-anti-rabbit IgG (Sigma-Aldrich) in PBST, after which they were placed in a humid container and allowed to incubate overnight in the dark at 4 °C. The slides were once again washed three times in PBST and dried, and cover slips were mounted using 10% glycerol solution containing 0.1 M propyl gallate. The sections were visualised under a Leica DM2500 LED optical fluorescent microscope (Leica Microsystems, Wetzlar, Germany).

Adult *F. hepatica* E/S and somatic extract (10 μg/lane) were resolved by gel electrophoresis in 4–20% SDS-PAGE gels and electro-transferred onto nitrocellulose membranes prior to incubation in blocking buffer for 1 h at RT. The membranes were probed overnight at 4 °C with rabbit pre-immune sera, anti-rFhSOD1, or anti-rFhSOD3 at a 1:1000 dilution. After washing, the membranes were further probed with the secondary antibody alkaline phosphatase conjugated goat-anti-rabbit IgG at a 1:10,000 dilution for 1 h at RT. Following final washes, the immune-reactive bands were visualised using the substrate SigmaFast BCIP/NBT (Sigma-Aldrich).

### 2.9. Killing of F. hepatica NEJ with Superoxide and Protection with SOD and Catalase

To determine the susceptibility of *F. hepatica* NEJ to superoxide in vitro, the SOD activity assay described in Section 2.5 was repurposed to examine the effect of enzymatically generated superoxide on live NEJ. In this application, assay conditions and enzyme concentrations, except for catalase (CAT, recombinant from Serratia; Abcam, Cambridge, UK) and BS, were as described above in Section 2.5. Tetrazolium salt was omitted from the assay buffer (AB) to avoid any potential toxicity to the NEJ. *F. hepatica* metacercariae were excysted as described above, washed in 1 × PBS and incubated as described below.

At total of 10 NEJ per replicate were incubated in (i) PBS; (ii) AB with XOD; (iii) AB with XOD and BS (0.16 U/mL); (iv) AB with XOD and CAT (43 U/mL); (v) AB with XOD, BS and CAT (43 U/mL); (vi) AB with XOD and CAT (4.3 U/mL); and (vii) AB with XOD, BS and CAT (4.3 U/mL). NEJ were incubated at 37 °C and 5% CO_2_ immediately after addition of XOD. After 24 h incubation, the number of dead NEJ (characterised by a lack of movement, including the absence of gut activity or a complete breakdown of the tegument and internal structure, after a one-minute observation) were counted. All treatments were conducted in duplicate over several days, giving a total of six biological replicates per treatment. To test the capacity of our recombinant enzymes to counteract the impacts of ROS on NEJ, we repeated the assay with the inclusion of rFhSOD1 and rFhSOD3 at concentrations of equal enzyme activity to BS, with and without the addition of catalase.

### 2.10. Statistical Analysis

Data were collected, stored, and analysed in Microsoft Excel version 16, Microsoft, Corporation, Redmond, WA, USA and GraphPad Prism version 5, GraphPad, San Diego, CA, USA. Differences between treatment groups were assessed by one-way ANOVA and Tukey’s post hoc test with 95% confidence intervals.

## 3. Results

### 3.1. The F. hepatica Genome Contains Developmentally Regulated Cytosolic and Extracellular SODs

**Figure 1:**
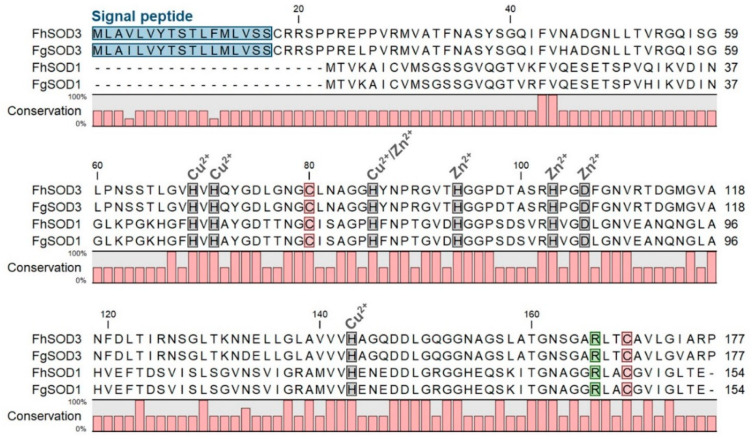
Comparative analysis of *F. hepatica* and *F. gigantica* SOD1 and SOD3 amino acid sequences. Metal binding sites are highlighted in grey and predicted signal peptides in blue. The arginine residue responsible for guiding the superoxide anion into the active site is indicated in green and the cysteine residues involved in disulphide bond formation are shown in red. Gaps are indicated by dashes. Amino acid conservation is shown below the alignment.

Analysis of the *F. hepatica* genome identified three superoxide dismutase genes, corresponding to the three types of SOD molecules identified within helminth parasites. Here, we report the two SODs that are known to be secreted by *F. hepatica* and, therefore, act at the host-parasite interface; a cytosolic SOD referred to herein as FhSOD1; and an extracellular SOD identified according to the presence of an N-terminal signal peptide sequence referred to herein as FhSOD3 (Figure 1). FhSOD1 corresponds to the previously reported cytosolic SOD [15,16].

**Figure 2:**
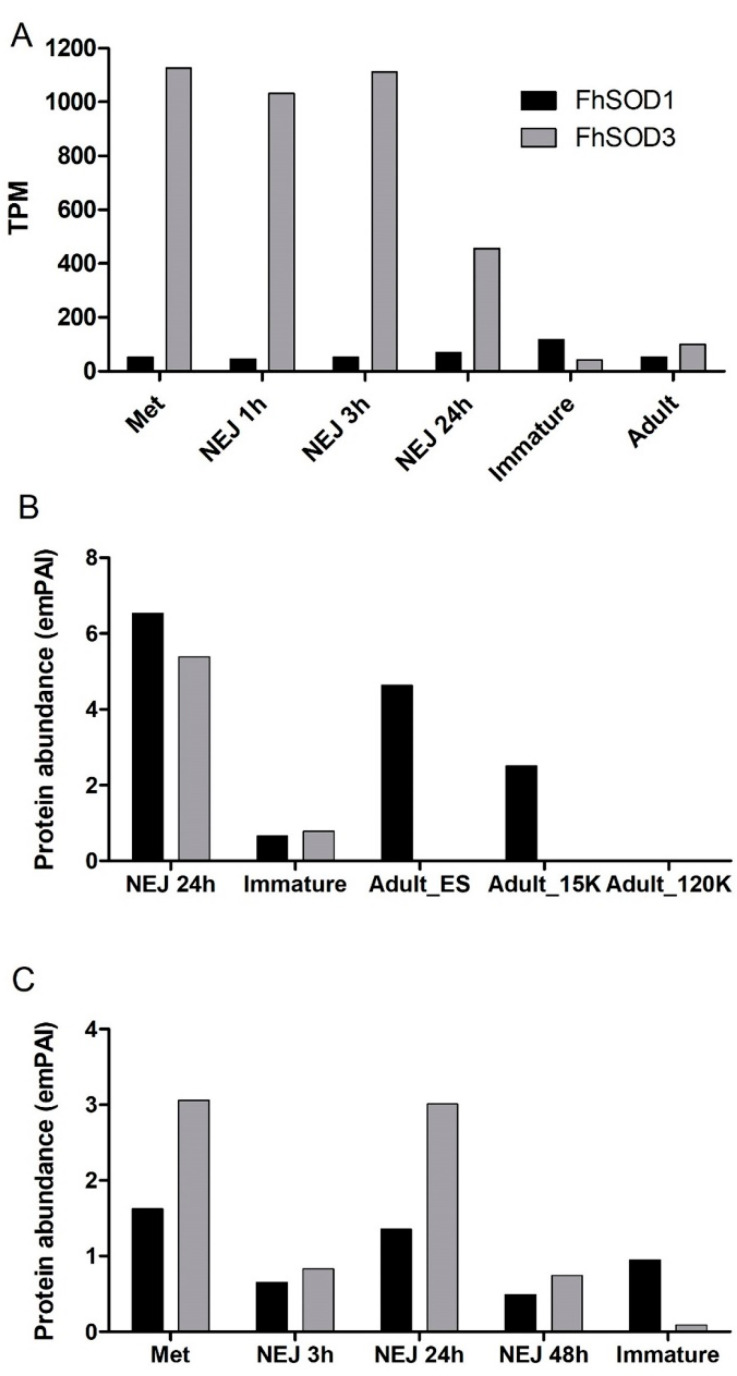
Expression profile of FhSOD1 and FhSOD3 throughout the *F. hepatica* life cycle within the mammalian host. (**A**) Graphical representation of the stage-specific transcription of the FhSOD1 and FhSOD3 genes displayed as transcripts per million (TPM) extrapolated from the transcriptome study of the metacercariae (Met), newly excysted juveniles at 1, 3, and 24 h post-excystment (NEJ 1 h, NEJ 3 h, NEJ 24 h), immature and adult liver flukes by Cwiklinski et al. [5]. (**B**) Graphical representation of the FhSOD1 and FhSOD3 proteins secreted within the ES protein fraction by the NEJ, immature, and adult parasites, represented by the exponentially modified protein abundance index (emPAI). The adult secretome data are displayed as the EV-depleted fraction (Adult_ES), and the microvesicle (Adult_15K) and exosome (Adult_120K) sub-fractions of the EV component recovered following centrifugation at 15,000× *g* and 120,000× *g*, respectively. (**C**) Graphical representation of the FhSOD1 and FhSOD3 protein abundance within the somatic proteome of the metacercariae (Met); newly excysted juvenile (NEJ) 3, 24, and 48 h post-excystment (NEJ 3 h; NEJ 24 h; NEJ 48 h); and immature liver flukes. The proteomic data for the NEJ, immature, and adult parasites are extrapolated from Cwiklinski et al. [6], Cwiklinski et al. [7], and Murphy et al. [30], respectively.

The two SOD genes display different transcriptional profiles across the developmental parasitic stages found within the mammalian host (Figure 2A). FhSOD1 is constitutively expressed at low levels across all the life cycle stages analysed. In contrast, FhSOD3 displays a markedly higher level of transcription by the infective stage metacercariae and the NEJ, which then drops to comparable levels to FhSOD1 expression within the immature and adult flukes.

Despite the relatively lower level of gene transcription, proteomic analysis revealed that FhSOD1 is more abundantly secreted than its extracellular counterpart, FhSOD3, predominantly by the NEJ and adult parasite stages (Figure 2B). Analysis of the adult secretome revealed that, in addition to being secreted within the protein soluble fraction, FhSOD1 is also found within extracellular vesicles (EVs), specifically the microvesicles that are known to be released by the gastrodermal cells of the gut. This is consistent with other *F. hepatica* proteins that lack a signal peptide sequence for classical secretion, highlighting that the parasite uses non-classical routes of secretion to release multiple molecules that interact with its host [31]. FhSOD3 is abundantly secreted by the NEJ parasites consistent with its gene transcription profile, with low levels secreted by immature flukes and no protein detected within the adult stages (Figure 2B). The somatic proteome profile for both FhSOD proteins is also consistent with that observed at the gene level, with comparable levels of FhSOD1 being detected across all the life cycle stages analysed, in comparison to FhSOD3, which displays a higher protein abundance within the metacercariae and NEJ 24 h post-excystment (Figure 2C).

### 3.2. Cytosolic and Extracellular SODs Are Distinct Yet Highly Conserved in Trematodes

Interrogation of available Platyhelminth genomes revealed a number of homologous SOD1 and SOD3 genes (SOD1: n = 14; SOD3: n = 19) from parasitic flatworms of class Trematoda and free-living flatworms of class Turbellaria. Phylogenetic analysis separates the SOD1 and SOD3 sequences into two distinct clades (Figure S2). The *F. hepatica* SOD1 and SOD3 sequences cluster with homologous sequences from F. gigantica and Echinostoma caproni, as is expected in these closely related parasite species. The majority of the SOD genes are present within their respective genomes as single copy genes, with the exception of Paragonimus westermani, which appears to contain two SOD1 sequences, while several *Schistosoma* spp. appear to contain two SOD3 sequences that cluster into distinct sub-clades (Figure S2). The SOD1 sequence from the free-living flatworm *Schmidtea mediterranea* does not cluster with its parasitic counterparts within the SOD1 clade.

All the SOD1 sequences analysed were comparable with that observed for the *F. hepatica* and *F. gigantica* SOD1 sequences, in that they all lacked an N-terminal signal peptide for classical secretion in line with the predicted cytosolic function of these proteins (data not shown). The majority of the Platyhelminth SOD3 sequences contain a signal peptide implying a common extracellular role for these proteins. Three Platyhelminth SOD3 sequences were missing the signal peptide sequence in the current respective genome assemblies (*E. caproni* SOD3, *T. regenti* SOD3, *S. rodhaini* SOD3; Table S1), with further investigation required to confirm their annotation.

Alignment of FhSOD1 with FhSOD3 revealed 34.64% sequence identity and 63.28% sequence similarity (ClustalO/uniprot) (Table S2). Comparison with the predicted *F. gigantica* SOD1 and SOD3 amino acid sequences shows a high level of conservation between the two species, with 98.70 and 100.00% identity and similarity, respectively, between the SOD1 sequences, and 96.61 and 99.44% identity and similarity, respectively, between the SOD3 sequences (Table S2). FhSOD1 has an average sequence identity of 69.81% to other Platyhelminth SOD1 sequences, and an average sequence identity of 59.42% with the four selected mammalian (*O. aries*, *B. taurus, B. indicus*, and *H. sapiens*) SOD1 sequences (Table S2). The trematode SOD1 sequences showed an average similarity of 90.20%, whereas the SOD3 sequences were more diverse, with an average similarity of 68.40% (Table S2). Collectively, this information suggests that FhSOD1 is more closely related to the human cytosolic enzyme than its own extracellular enzyme. Alignment against the respective *O. aries*, *B. taurus*, *B. indicus*, and *H. sapiens* SOD1 and SOD3 sequences shows 100% conservation of predicted copper and zinc metal binding sites (His69, His71, His86, His94, His103, Asp106, His143) (Figure S1; residue numbering relative to FhSOD3) [11,14,16].

### 3.3. Recombinant FhSOD1 and FhSOD3 Are Highly Active

**Figure 3:**
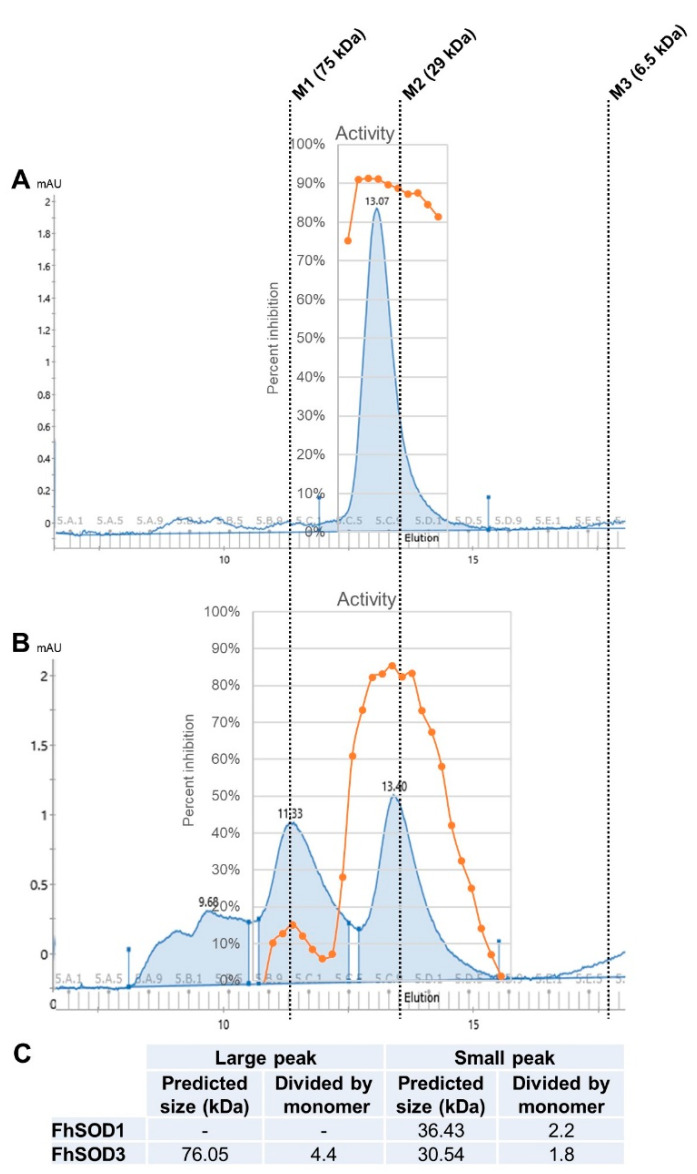
Structural organisation and enzymatic activity of rFhSOD1 and rFhSOD3. Size exclusion chromatography of (**A**) FhSOD1 and (**B**) FhSOD3. The corresponding enzyme activity of each elution fraction is expressed as the percentage inhibition of formazan dye formation. (**C**) The predicted molecular sizes of each recombinant protein calculated against the molecular weight markers. Markers are indicated by dotted lines—M1; conalbumin, M2; carbonic anhydrase, M3; aprotinin.

To investigate the role of *F. hepatica* SODs in parasite metabolism and host defence, we exploited E. coli to recombinantly express both FhSOD1 and FhSOD3 antioxidant proteins. SDS-PAGE and Western blot analysis using monoclonal anti-histidine antibodies revealed soluble rFhSOD1 and rFhSOD3 purified at ~16 and ~17 kDa, respectively, which are predicted to have a conserved secondary structure consisting of a β-sheet made up of eight antiparallel β-strands typical of SODs (Figures S3 and S4). rFhSOD1 and rFhSOD3 had similar activity against superoxide (~400 U/mg each, as defined by the standard curve), where one unit of enzyme activity is defined as the amount of protein required to exhibit 50% dismutation of superoxide under physiological conditions (150 mM salt, pH 7.4, 37 °C), albeit they were ~10 times less active than the positive control (Figure S3). Size exclusion (gel filtration) chromatography revealed distinct peaks corresponding to molecular weights of ~36 kDa (rFhSOD1), ~31 kDa (rFhSOD3), and ~76 kDa (rFhSOD3), indicative of homodimers and a mix of homodimers and homotetramers for rFhSOD1 and rFhSOD3, respectively (Figure 3). Analysis of the individual fractions collected from each peak demonstrate that both the dimeric (rFhSOD1 and rFhSOD3) and tetrameric (rFhSOD3) forms of the recombinant proteins are enzymatically active (Figure 3).

### 3.4. Detection of Native F. hepatica SOD

**Figure 4:**
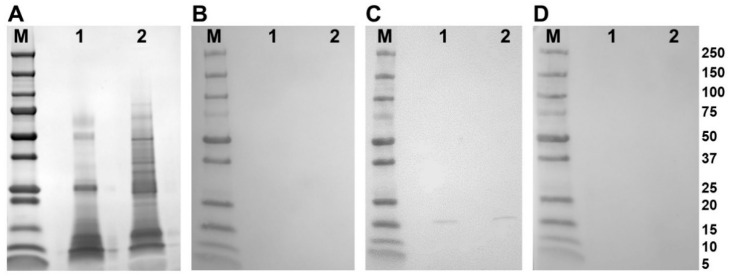
Immune detection of native *F. hepatica* SODs in adult parasite E/S and somatic extracts. (**A**) Native (10 ug/well) *F. hepatica* adult worm E/S and somatic extracts were resolved in a 4–20% SDS-PAGE gel and stained with Biosafe Coomassie. Lane 1: adult E/S; lane 2: adult somatic extract. (**B**–**D**) Western blot analysis of native FhSOD1 and FhSOD3. Lane 1: adult E/S; lane 2: adult somatic extract. Immuno blots were probed with (**B**) rabbit pre-immune sera (negative control), (**C**) anti-rFhSOD1 polyclonal antibodies raised in rabbit, and (**D**) anti-rFhSOD3 polyclonal antibodies raised in rabbit. M molecular weight in kDa (Precision Plus Protein Dual, Bio-Rad).

**Figure 5:**
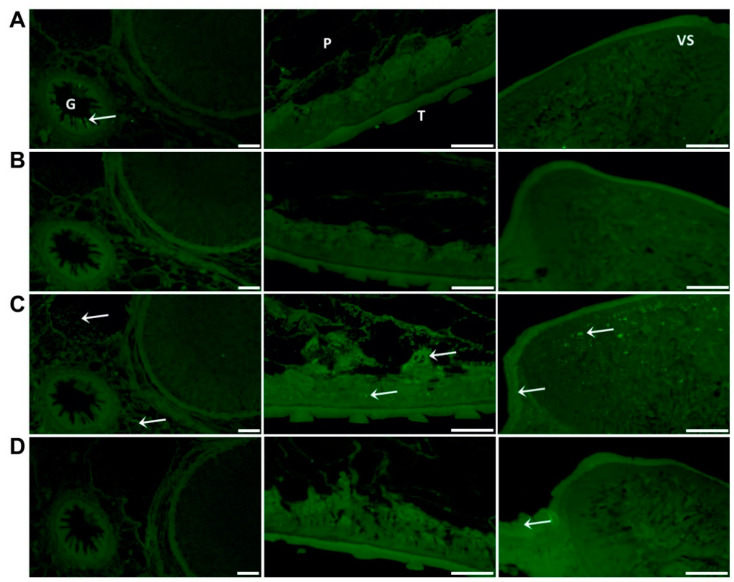
Immuno-localisation of native FhSOD1 and FhSOD3 in *F. hepatica* adult sections. Sections were probed with (**A**) anti-rFhCL1 polyclonal antibodies (non-related positive control), (**B**) rabbit pre-immune sera (negative control), (**C**) anti-rFhSOD1 polyclonal antibodies, and (**D**) anti-rFhSOD3 polyclonal antibodies raised in rabbit. Immuno-localisation of native *F. hepatica* proteins is represented by green fluorescence (FITC staining) and indicated with white arrows. G; gut, P; parenchyma, T; tegument, VS; ventral sucker, scale bars 50 µM.

Western blot analysis of adult parasite E/S and somatic extract using polyclonal antibodies raised in rabbits to the recombinant FhSODs revealed detectable FhSOD1 in both extracts at the expected size (Figure 4). In agreement with transcriptome and proteome data from adult parasites, native FhSOD3 was not detected in either fraction by polyclonal antibodies against rFhSOD3. These results are consistent with the immuno-detection of the proteins in adult parasite sections, where FhSOD1 was distributed throughout the muscle, tegument, and parenchyma, while limited FhSOD3 was visible in the tegument (Figure 5). The anti-pFhSOD1 and pFhSOD3 antibodies demonstrated protein-specific binding against rFhSOD1 and rFhSOD3 by Western blot analysis but did not reveal detectable fluorescence when applied to fixed parasite specimens, suggesting recognition of a linear epitope in NEJ (Figure S5).

**Figure 6:**
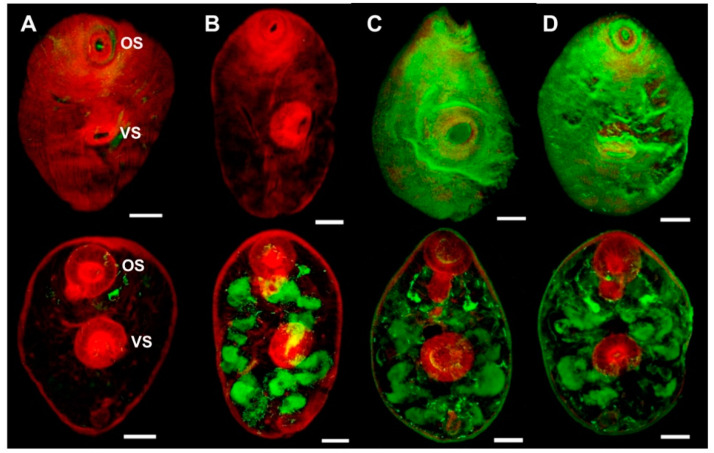
Immuno-localisation of native FhSOD1 and FhSOD3 in newly excysted juveniles (NEJ). Whole-mount *F. hepatica* NEJ 3 h post-excystment were probed with (**A**) rabbit pre-immune sera (negative control), (**B**) anti-rFhCL3 polyclonal antibodies (non-related positive control), (**C**) anti-rFhSOD1 polyclonal antibodies, and (**D**) anti-rFhSOD3 polyclonal antibodies. Immuno-localisation of native *F. hepatica* proteins is represented by green fluorescence (FITC staining). All samples were counter-stained with phalloidin-TRITC to stain muscle tissue (red fluorescence). OS; oral sucker, VS; ventral sucker, scale bars; 25 µM.

Whole mount immuno-localisation of NEJ 3 h post-excystment using anti-rFhSOD1 and anti-rFhSOD3 antibodies revealed the presence of FhSOD1 and FhSOD3 on the outer surface, gut and tegument as indicated by diffuse fluorescence (Figure 6). This is in contrast to NEJ stained with anti-rFhCL3 polyclonal antibodies, which locates the FhCL3 cysteine peptidase solely within the bifurcated gut (Figure 6). Despite a potential cross-reaction between the two FhSOD polyclonal antibodies, these results are consistent with the transcriptome and proteome analyses of the juvenile parasites, which show abundant expression by the metacercariae and 1, 3, and 24 h post-excystment NEJ (Figure 2A,C).

### 3.5. Homology with Host SOD Facilitates Immune Evasion

**Figure 7:**
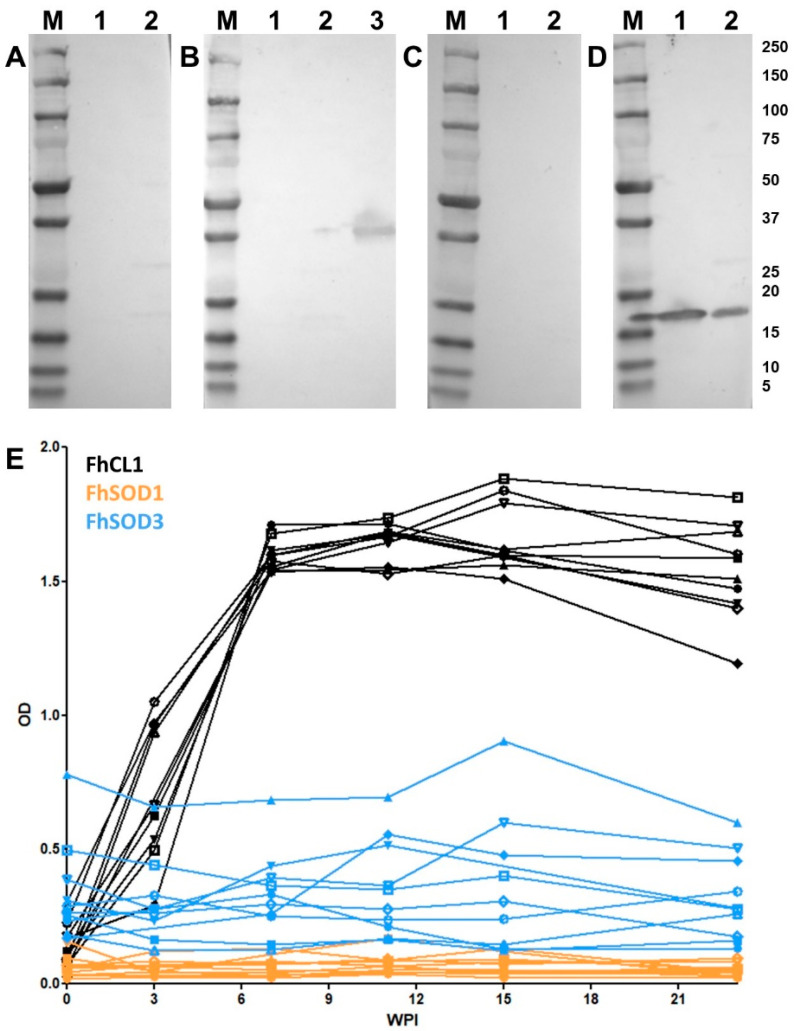
Antibody responses to recombinant FhSOD1 and FhSOD3 in experimentally infected sheep. (**A**–**C**) Recombinant (1 ug/well) FhSOD1 and FhSOD3 were transferred to a nitrocellulose membrane and probed with pooled sera from experimentally infected sheep (**A**) pre-infection (negative control), (**B**) 7 weeks post-infection (WPI), and (**C**) 20 WPI. (**D**) Recombinant FhSOD1 and FhSOD3 were probed with anti-SOD1 and anti-SOD3 polyclonal antibodies raised in rabbits (positive control). Lane 1; recombinant FhSOD1, lane 2; recombinant FhSOD3, lane 3; recombinant FhCL1 zymogen mutant (positive control), M; molecular weight in kDa (Precision Plus Protein Dual, Bio-Rad. (**E**) The optical density of IgG antibodies to rFhSOD1, rFhSOD3 and rFhCL1 from experimentally infected sheep 0, 3, 7, 11, 15 and 25 WPI.

Western blot analysis of rFhSOD1 and rFhSOD3 probed with pooled sera from experimentally infected sheep demonstrated that these proteins are not immunogenic during *F. hepatica* infection (Figure 7A–D). These results were confirmed by analysis of individual sheep sera collected at 0, 3, 7, 11, 15, and 23 WPI by ELISA against rFhSOD1 and rFhSOD3, using the immunogenic cathepsin peptidase, rFhCL1, as a positive control (Figure 7E).

### 3.6. The Addition of SOD and Catalase Negate the Lethal Effects of Reactive Oxygen Species against F. hepatica NEJ In Vitro

**Figure 8:**
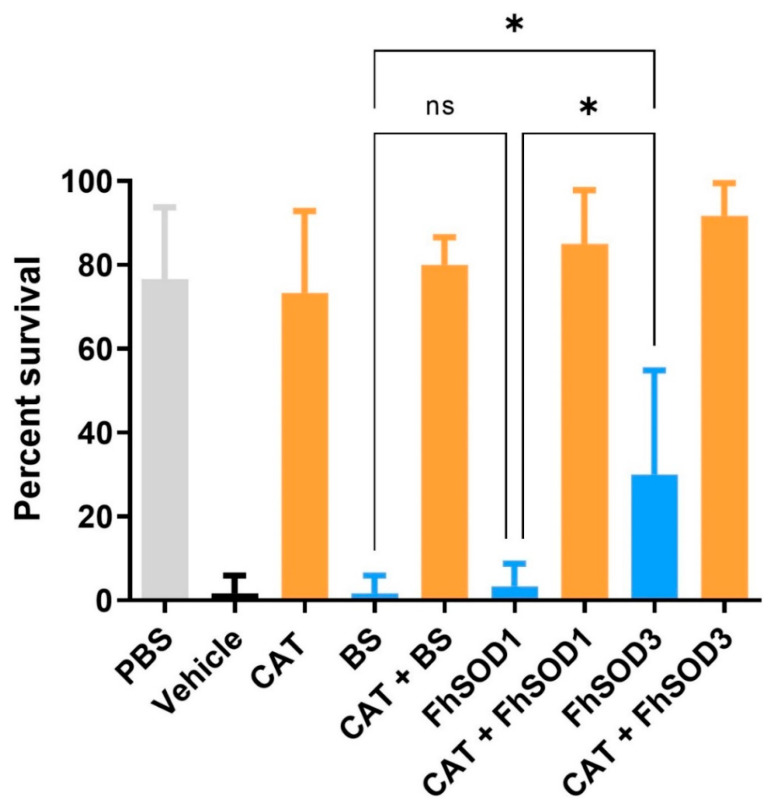
Susceptibility of *F. hepatica* NEJ to superoxide and their subsequent protection via the addition of SOD and catalase. Six replicates of 10 NEJ/well were incubated in the presence of enzymatically generated superoxide for 24 h at 37 °C and 5% CO_2_. PBS; phosphate buffered saline (negative control), Vehicle; assay buffer containing enzymatically generated superoxide via the step-wise reduction of hypoxanthine into xanthine and superoxide by xanthine oxidase (positive control), CAT; recombinant catalase, BS; native bovine erythrocyte SOD, ns; not significant (0.1234), * *p* < 0.0332.

Co-incubation of *F. hepatica* NEJ with enzymatically generated superoxide killed 98% of exposed NEJ within 24 h. There was no significant difference in NEJ survival after the addition of BS or rFhSOD1 (*p* > 0.99); however, rFhSOD3 prevented death in 30% of NEJ (*p* = 0.0041) (Figure 8; Table S3). The addition of catalase protected NEJ in a dose-dependent manner, with 73 and 43% of NEJ alive when incubated with 43 and 4.3 U/mL catalase, respectively, at 24 h (Figure S6), the effect of which was enhanced when used in the conjunction with SODs (Table S3).

## 4. Discussion

Superoxide dismutases (SODs) are amongst a collection of antioxidants that play essential roles in parasite defence against oxygen free radicals generated physiologically by cellular metabolism and externally by innate immune cells, such as macrophages and neutrophils, during invasion and infection [4,7,32]. Similar to many trematodes, the liver fluke *F. hepatica* possesses a complex system involved in host immuno-modulation and immuno-evasion wherein a repertoire of proteins is excreted/secreted to simultaneously defend against and distract the host’s immune response [30,31]. By interrogating existing genomic, transcriptomic, and proteomic analysis of various intra-mammalian life stages of *F. hepatica,* we identified several antioxidants in the protein cocktail deployed by the parasite during the early invasive and migratory processes, including both a cytosolic (FhSOD1) and an extracellular (FhSOD3) SOD [5,6,7]. Identified and characterised in *F. hepatica* for the first time herein, we demonstrate that FhSOD3 is NEJ-associated and, thus, likely to play a key role in invasion of the mammalian host; for example, the penetration of the intestine and liver tissue. FhSOD1 on the other hand, which is released into host tissues through alternative secretion pathways, may function simultaneously in parasite metabolism and defence against exogenous ROS. Through a series of immuno-localisation, serological, and functional in vitro experiments, we propose that FhSOD1 and FhSOD3 play distinct roles in the development of *F. hepatica* and its interaction with mammalian definitive hosts.

As is observed with other proteins excreted/secreted by *F. hepatica* throughout its life cycle, such as the cathepsin L and B proteases, the transcription and expression of FhSOD1, and FhSOD3 are tightly regulated [6,7]. FhSOD1 is constitutively transcribed across all *F. hepatica* life stages at relatively low levels. FhSOD3, however, is highly expressed in the infective metacercariae and early NEJ, before declining to transcription levels, similar to FhSOD1 in immature and adult worms. The lower level of FhSOD1 transcription in metacercariae and NEJ compared to FhSOD3 is in contrast to the relative protein abundance in these early stages and suggests that the parasite produces and stores FhSOD1 prior to excystment in the small intestine. Both FhSOD1 and FhSOD3 are at their most abundant in *F. hepatica* E/S and somatic extract early in the life cycle, which implies that they are vital during early invasion of the mammalian host. Macrophages and neutrophils play an essential role in the early innate immune response against invading pathogens via the production of ROS, and thus it is reasonable to suggest that *F. hepatica* NEJ, which are vulnerable and living on limited glycogen stores, would have a pre-prepared store of antioxidants to defend against this onslaught of ROS [33]. The abundance of both FhSODs in metacercariae and NEJ may also reflect a defence against ROS arising from normal metabolic processes occurring in the most environmentally robust stage of the parasite.

Of the two proteins, only FhSOD3 possesses an N-terminal signal peptide and is, thus, transported to the extracellular environment via the classical secretory pathway. FhSOD1, on the other hand, is found in both the microvesicular fraction and the EV-depleted supernatant of adult *F. hepatica* E/S. This is an interesting finding given that cytosolic SOD enzymes are thought to act solely on endogenous ROS produced during cellular metabolism, and not interact with host-derived ROS [32]. Although the mechanism(s) by which this compartmentalisation of FhSOD1 occurs remains unknown, the presence of FhSOD1 in different vesicular and non-vesicular fractions of the E/S suggests distinct methods of delivery to host immune cells and, thus, different defensive or immuno-modulatory roles. Given the observed homology between the cytosolic SOD of *F. hepatica* and other trematode parasites, it is worth investigating if these findings are unique to this species or if they are indicative of an as-of-yet unexplored generalised helminth defence mechanism. Contrary to the cytosolic SOD, the diversity observed between the signal-peptide containing extracellular SOD, including the apparent duplication of this isoform within *Schistosoma* spp., could indicate unique host-specific adaptations in these worms.

In the current study, neither rFhSOD1 nor rFhSOD3 were immunogenic in experimentally infected sheep. Our findings reflect those previously observed when both experimentally and naturally infected buffalo sera were probed for antibodies to FgSOD1 [17]. Similarly, there has been a lack of detectable antibodies when sera from experimentally infected rats were probed with recombinant serine protease inhibitors (serpins; rFhSrp1/2), and when sera from experimentally infected sheep was probed with recombinant cathepsin B1 (rFhCB1) [28,29]. FhSOD1 is highly homologous to its mammalian counterpart, with an average similarity of 87.34% compared to SOD1 sequences from *O. aries, B. taurus, B. indicus*, and *H. sapiens*. It is possible that the parasite exploits these similarities as yet another strategy of immune evasion, wherein their high host homology and, thus, low immunogenicity allows them to operate undetected and unimpeded. Indeed, this host mimicry has been observed in *Schistosoma mansoni*, another trematode parasite of humans, leading to concerns that this high homology with host molecules may induce autoimmune responses during vaccine trials [34].

Unlike *F. hepatica*, the cytosolic SOD of *S. mansoni* (SmCT-SOD) is expressed at the highest levels in adult worms, leaving the larval stages susceptible to immune elimination via host-generated superoxide [34]. In these parasites, SmCT-SOD was localized to the tegument and gut epithelium of adults. This contrasts with the findings of the current study, which showed that FhSOD1 and FhSOD3 localise to the tegument and gut of NEJ and show a marked decline in FhSOD-specific immuno-localisation in adult parasites. The differential life-stage expression of cytosolic SOD between *F. hepatica* and *S. mansoni* may reflect different host migration routes and tissue tropism between these parasites. *S. mansoni* infects mammalian hosts via penetration of the skin before eventually residing in the mesenteric venules, where they would be more available to host-generated ROS than *F. hepatica*, which is located within the bile ducts and gallbladder upon maturation [35]. Nevertheless, in both parasites, immuno-localisation occurs at the host:parasite interface and supports the theory that these proteins are employed in defence of exogenous superoxide.

Previous studies have shown that *F. hepatica* NEJ are resistant to killing by superoxide produced in vitro chemically or by peritoneal lavage cells isolated from Indonesian Thin-Tailed (ITT) sheep [36,37]. ITT sheep are resistant to infection by *F. gigantica* but have been shown to be susceptible to infection with *F. hepatica* [38]. It was suggested that this susceptibility is related to the higher gene expression of SOD1 mRNA in *F. hepatica* NEJ compared to *F. gigantica* [36]. In the current study, we demonstrated robust in vitro killing of *F. hepatica* NEJ by enzymatically generated ROS. Interestingly, ROS-associated killing of NEJ was partially prevented via the addition of rFhSOD3 only, but completely inhibited via the introduction of catalase. Given that all three SODs were utilized at concentrations of equal enzyme activity, these results suggest that rFhSOD1 and rFhSOD3 do not behave similarly under physiological conditions. Further, the production of hydrogen peroxide in our assay results from the (a) two-step reduction of hypoxanthine into xanthine and superoxide and (b) the reduction of superoxide by SOD. It is well known, however, that the production of superoxide by macrophages during the oxidative burst is part of a cascade of highly damaging reactive oxygen and nitrogen species; thus, our data imply that *F. hepatica* needs to employ an array of antioxidant proteins to defend against cell-mediated immune responses [9,32]. Helminths do not express catalase, but rather exploit a thiol-independent antioxidant system to detoxify hydrogen peroxide wherein thioredoxin/glutathione reductase (FhTGR) reduces thioredoxin (FhTrx), which then reduces and activates peroxiredoxin (FhPrx), all of which are up-regulated in *F. hepatica* NEJ [7,8,39]. In our in vitro assay, we utilized catalase instead of FhPrx, and thus circumvented this complex cascade and provided proof-of-principal evidence that *F. hepatica* NEJ exploit a series of antioxidants to defend against host ROS during early invasion. Future work will focus on the complex interactions between each of these antioxidant proteins and their collective role in combatting damaging ROS.

## 5. Conclusions

We propose that *F. hepatica* exploits two distinct SODs to defend against host-generated ROS during early invasion and infection. We have shown that these proteins have unique expression profiles and secretory pathways, and are, thus, likely to play divergent roles in the development and growth of the parasite in its mammalian host. Going forwards, it will be imperative to define how these proteins interact with each other and with the slew of other antioxidant proteins secreted by the parasite during early invasion, and thus work in concert to detoxify ROS intracellularly and in the extracorporeal environment.

## Data Availability

The transcriptome data sets used to extrapolate the FhSOD gene transcription were previously reported by Cwiklinski et al. [5] and are available in the European Nucleotide Archive repository, PRJEB6904; http://www.ebi.ac.uk/ena/data/view/PRJEB6904 (30 August 2022). The mass spectrometry proteomics data analysed as part of this study have been deposited to the ProteomeXchange Consortium via the PRIDE partner repository with the following data set identifiers (a) NEJ specific datasets Cwiklinski et al. [6]: PXD007255, PXD016561; (b) immature fluke Cwiklinski et al. [7]: PXD021221; (c) adult ES and EV datasets Murphy et al. [30]: PXD002570 and PXD016561.

## Supplementary Materials

The following supporting information can be downloaded at: https://www.mdpi.com/article/10.3390/antiox11101968/s1, Table S1. Trematode and mammalian amino acid sequence details for phylogenetic analysis. Table S2. Similarity and identity of trematode and mammalian SOD1 and SOD3 amino acid sequences. Table S3. Significance of *F. hepatica* NEJ survival rates when co-cultured with enzymatically generated superoxide dismutase in vitro. Figure S1. Alignment of mammalian and trematode SOD1 and SOD3 amino acid sequences for phylogenetic analysis. Selection of conserved blocks from the complete SOD alignment was determined by Gblocks 0.91b [18,19], with 41% of the positions conserved. Metal binding sites are indicated by grey bars. Gaps are shown as dashes. Variable residues are highlighted in red. *Fasciola hepatica* and *Fasciola gigantica* sequences are highlighted in bold. The first amino acid is numbered 67 (relative to FhSOD3—see Figure 1 in main text). Figure S2. Phylogenetic analysis of trematode and mammalian SOD amino acid sequences. Phylogenetic analysis was carried out using the maximum likelihood method and the Whelan and Goldman model [21]. The tree with the highest log likelihood (−3031.18) is shown. The percentage of trees in which the associated taxa clustered together (out of 1000 iterations) is shown below the branches (Bootstrap support values less than 50% are omitted). The tree is drawn to scale, with branch lengths measured in the number of substitutions per site. Figure S3. Expression and enzyme activity of rFhSOD1 and rFhSOD3. (A) Recombinant proteins expressed in *E. coli* ClearColi cells and purified by affinity column, resolved in a 4–20% SDS-PAGE gel and stained with Biosafe Coomassie. (B) Recombinant proteins were electro-transferred onto a nitrocellulose membrane and probed with mouse monoclonal antibodies to poly-histidine (1:10,000). P; *E. coli* pellet, S; purified supernatant. M; molecular weight in kDa (Precision Plus Protein Dual, BioRad). (C) Activity of recombinant FhSOD1 and FhSOD3 compared to native bovine erythrocyte SOD (BS). Both rFhSOD1 and rFhSOD3 exhibited a specific activity of ~400 U/mg relative to the standard curve, where one unit is the amount of enzyme required to exhibit 50% dismutation of the superoxide radical. Figure S4. The predicted 3D structure of (A) FhSOD1 and (B) FhSOD3. Structure predicted by the AlphaFold Protein Structure Database [25,26]. The colour of each amino acid indicates the per-residue confidence score (pLDDT) where dark blue resembles very high confidence (> 90 pLDDT), light blue resembles confident (90 > pLDDT > 70), yellow indicates low confidence (70 > pLDDT > 50). Figure S5. Specificity of polyclonal and anti-peptide antibodies against recombinant *F. hepatica* SODs. (A) Purified recombinant (0.05 ug/well) FhSOD1 and FhSOD3 were resolved in a 4–20% SDS-PAGE gel and stained with Biosafe Coomassie. Lane 1: BS; lane 2: rFhSOD1; lane 3: rFhSOD3. (B–F) Western blot analysis of rFhSOD1 and rFhSOD3. Lane 1: BS; lane 2: rFhSOD1; lane 3: rFhSOD3. Immuno blots were probed with (B) rabbit pre-immune sera (negative control), (C) anti-FhSOD1 peptide antibodies, (D) anti-FhSOD3 peptide antibodies, (E) anti-rFhSOD1 polyclonal antibodies, and (F) anti-rFhSOD3 polyclonal antibodies raised in rabbit. Figure S6. SOD and catalase inhibit the lethal effects of superoxide and hydrogen peroxide on *F. hepatica* NEJ in vitro. (A) Principal of the assay demonstrating the step-wise reduction of hypoxanthine into xanthine and superoxide by xanthine oxidase and the subsequent production of hydrogen peroxide and uric acid as a by-product of the reaction. (B) Enzyme activity of rFhSOD1 and rFhSOD3 vs. BS prior to their inclusion in the NEJ assay at 0.01 mg/mL (rFhSODs) and 0.001 mg/mL (BS) (C) Killing of *F. hepatica* NEJ using enzymatically generated superoxide is prevented via the addition of catalase (CAT) and BS in a dose-dependent manner. PBS; negative control, Vehicle; positive control (superoxide generated in vitro). (D) The impact of superoxide and hydrogen peroxide on *F. hepatica* NEJ after 24 h of co-culture is shown. Black scale bars; 100 µM, white scale bar; 50 µM, ns; not significant.
