# Supplementary figures and images for: Two Distinct Superoxidase Dismutases (SOD) Secreted by the Helminth Parasite Fasciola hepatica Play Roles in Defence against Metabolic and Host Immune Cell-Derived Reactive Oxygen Species (ROS) during Growth and Development
Source: Antioxidants (Basel). 2022 Sep 30;11(10):1968. doi: 10.3390/antiox11101968 (PMC9598480; doi:10.3390/antiox11101968)

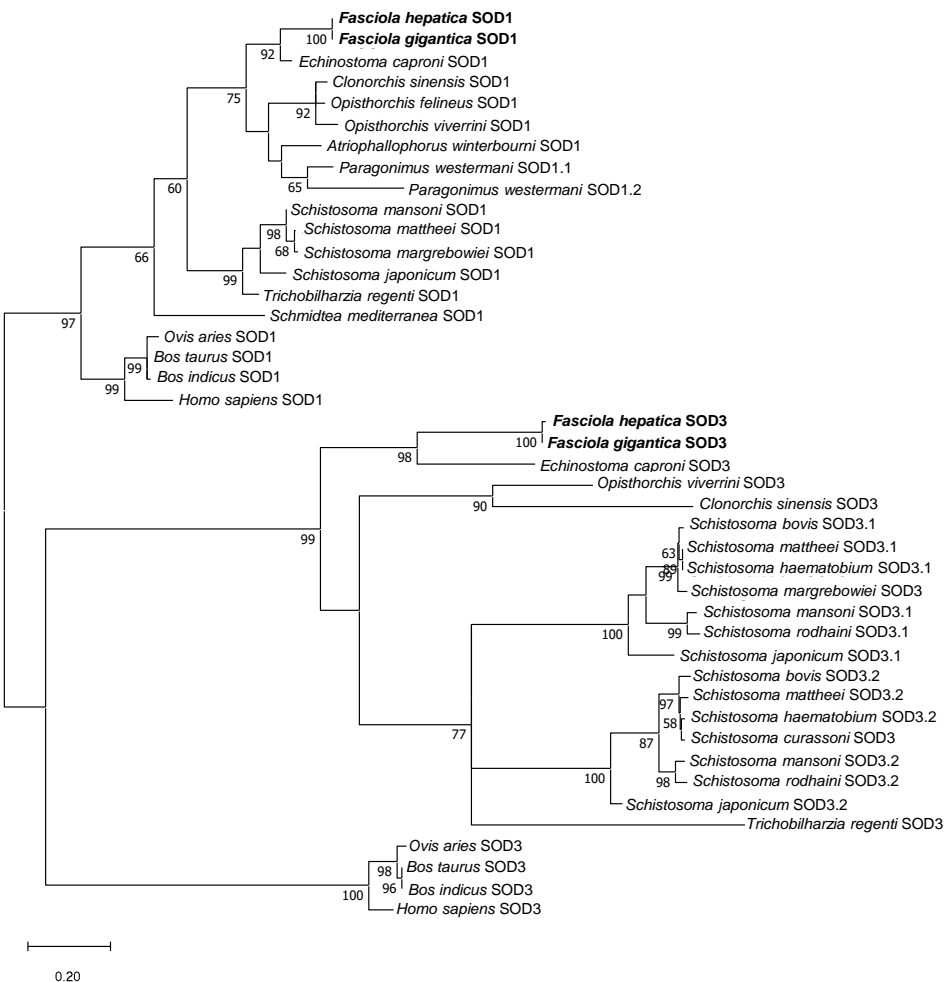

**Figure S2.**

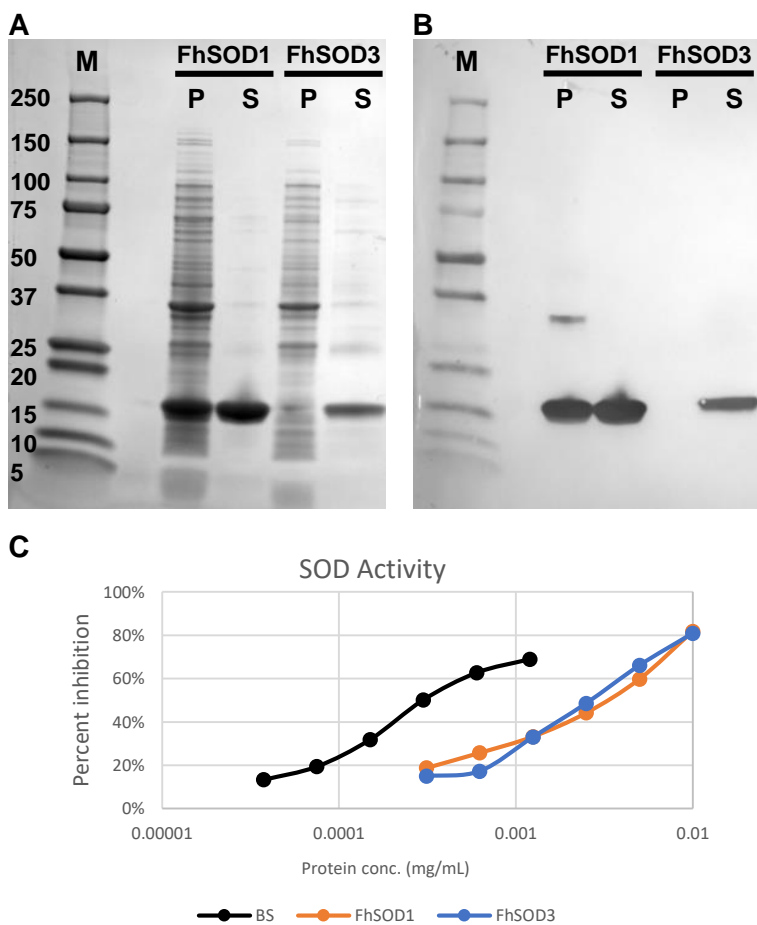

Figure S3.

**A**

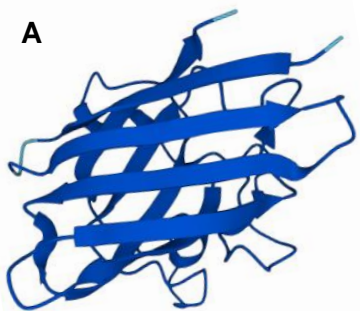

**B**

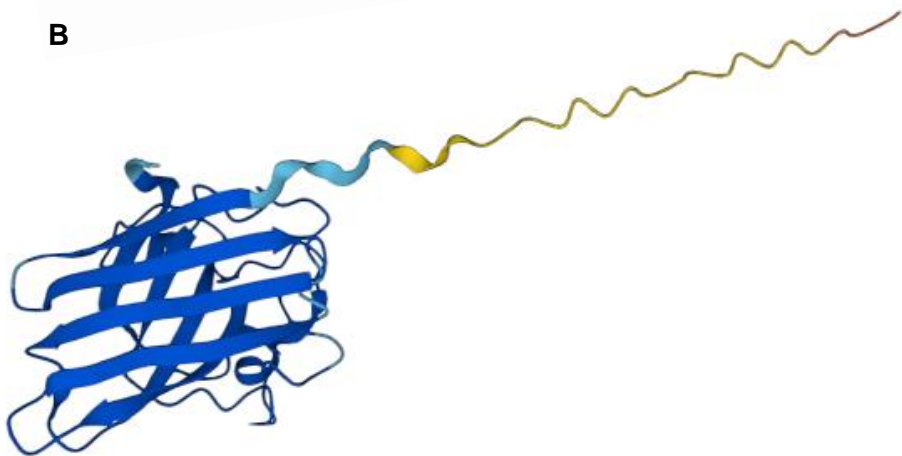

**Figure S4.**

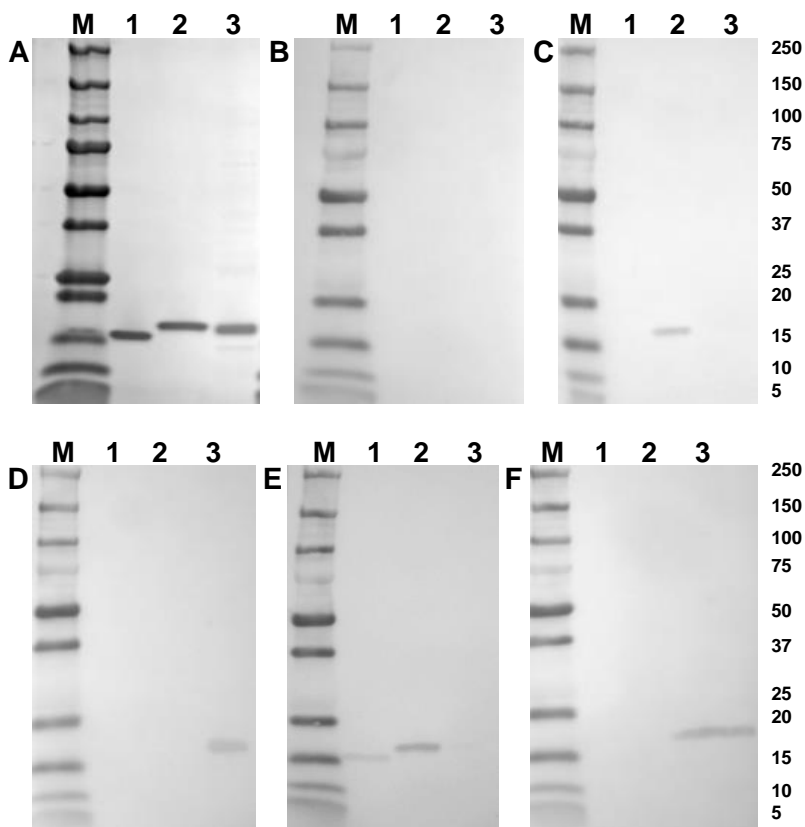

Figure S5.

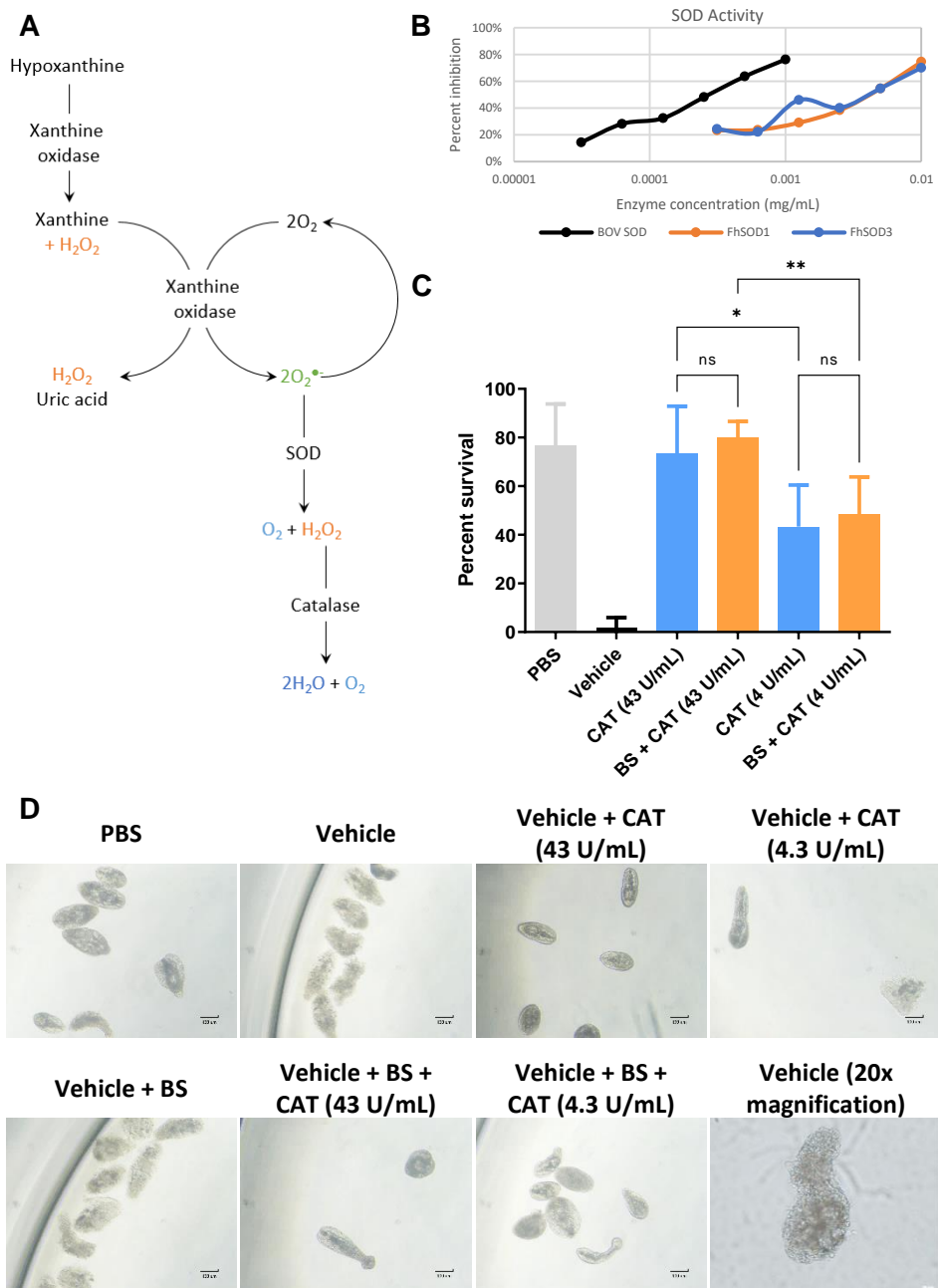

Figure S6.

Supplement: Supplementary file 1 [file antioxidants-11-01968-s001.zip › Figures_supp_R1.pdf]
